# Social Response to the Vaccine against COVID-19: The Underrated Power of Influence

**DOI:** 10.3390/jpm12010015

**Published:** 2021-12-29

**Authors:** Dimitra S. Mouliou, Ioannis Pantazopoulos, Konstantinos I. Gourgoulianis

**Affiliations:** 1 Department of Emergency Medicine, Faculty of Medicine, University of Thessaly, BIOPOLIS, 41110 Larissa, Greece; pantazopoulosioannis@yahoo.com; 2 Department of Respiratory Medicine, Faculty of Medicine, University of Thessaly, BIOPOLIS, 41110 Larissa, Greece; kgourg@uth.gr

**Keywords:** COVID-19, SARS-CoV-2, vaccination, vaccine

## Abstract

Background: The Coronavirus Disease 2019 (COVID-19) pandemic has highlighted the need for preventive medicine and vaccinology to be paralleled to eliminate COVID-19 cases. Methods: A web-based questionnaire was disseminated through social media in the late November assessing the factors that may have influenced the final response to vaccination against COVID-19 in vaccinated and non-vaccinated Greek people. Results: Women, the younger generations, and university graduates were more likely to accept vaccination, whereas men, those with a basic education level, and the older generation showed a hesitance to the vaccine against COVID-19. About half of the vaccinated participants were influenced in their final decision mainly by being informed from the internet (50.4%), their work (51.7%), and social life (53,1%) while half of the non-vaccinated individuals were mostly influenced by keeping updated from the internet (55.5%) and by government policies (51.3%). COVID-19 risk (OR 2.511; CI 2.149–2.934; *p* = 0.000), frequent vaccinations for emerging pathogens (OR 14.022; CI 11.998-16.389), and social life (OR 2.828; CI 2.417–3.309; *p* = 0.000) had a significant impact on people’s positive response to vaccination against COVID-19. Conclusions: Monitoring and assessing the influence factors for the response to vaccination can be favourable strategies to further manage societal vaccination rates.

## 1. Introduction

In December 2019, a novel Severe Acute Respiratory Syndrome Coronavirus 2 (SARS-CoV-2) was identified from a cluster of cases of pneumonia in Wuhan, China [1]. On 30 January 2020, the World Health Organization (WHO) announced the Coronavirus Disease 19 (COVID-19) as a Public Health Emergency of International Concern, and a month and a half later, the COVID-19 epidemic was portrayed as a pandemic [2].

Since 2020, the development of various vaccines to reduce the risk for a severe COVID-19, has been expedited, as the increasing infection rates of COVID-19 worldwide have stimulated international alliances to urgently organize resources to make multiple vaccines on shortened timelines [3,4]. Heretofore, some vaccines against COVID-19 are in Phase III or have ended Phase III clinical trials with positive results [5]. A few of these new vaccines are approved for emergency use, while the Centers for Disease Control and Prevention (CDC) have recently expanded recommendations for booster shots [6]. Until now, 53.5% of the world population has received at least one dose of a COVID-19 vaccine [7]. Nowadays, Singapore records the higher percentage of fully vaccinated individuals, compared to Ethiopia, which records 1.2% of fully vaccinated people [7].

On 26 February 2020, the first COVID-19 case was reported in Greece, and, at present, the country is still facing the forth COVID-19 pandemic wave [8]. A recent Greek study estimated that 81.2% of the population-based sample presented underlying medical conditions that could lead to a severe COVID-19 infection [9]. Greece accomplishes a unique daily massive rapid antigen testing strategy for monitoring the virus and a focused daily information of the public for the total SARS-CoV-2 positive test results. Despite the risk for potential false test results in mass-testing, a Greek study has accurately shown that the catering/food sector was the most vulnerable to COVID-19 before the lockdown, via massive rapid antigen testing strategies [10,11]. Up to now, about 1/10 of the Greeks have passed COVID-19 [12].

The COVID-19 vaccination campaign in Greece began on 27 December 2020, in line with the EU Vaccination Days, in accordance with the Greek government’s vaccination operation dubbed “Eleftheria”. The first recipient to be vaccinated with Comirnaty (BioNTech/Pfizer) was a nurse, the second one was a nursing home resident, the third one was the president of the Hellenic Republic, the fourth one was the Greek Prime Minister, and the fifth one was the head of the Greek COVID-19 health committee. Until now, five vaccines are approved for use in the country: Nuvaxovid/Covovax (Novavax), Spikevax (Moderna), Comirnaty (BioNTech/Pfizer), Janseen (Johnson & Johnson) and VaxZevria (Oxford/AstraZeneca) COVID-19 vaccines.

As by 23 December 2021, 66.6% of the Greek populace were fully vaccinated against COVID-19 [13]. Similar rates for volition to COVID-19 vaccine were revealed from another Greek study [14]. In Greece, non-vaccinated people or those without a previous COVID-19 infection certificate are now prohibited from entering indoor places, such as restaurants, museums, theaters, cinemas, gyms, MICE venues (meetings, incentives, conferences, and exhibitions), and stadiums, even with a negative test result. Moreover, a negative test is required for them to enter retail stores, hair and beauty salons, cafes and bars—only for outdoor seating, some workplaces, schools and other educational institutions, and recently churches. Non-vaccinated healthcare workers and those without the previous infection certificate are not allowed to work. Moreover, nowadays, Greek authorities announced that for individuals aged 60 years and over who have not been vaccinated by 16 January 2022, they will face a recurring monthly fine of 100 euros.

The aim of this study was to identify potential factors that had an impact on the final decision regarding vaccination against COVID-19. This study not only assessed the influence factors for vaccinated people, but also for non-vaccinated people, respectively. In this manner, an online survey with a Web-based Questionnaire (WBQ) was performed. A final comparison of the factors with the higher influence between the vaccinated participants is presented.

## 2. Materials and Methods

### 2.1. WBQ Design

Although WBQs are currently being considered as a fluid form of observational, descriptive, and analytical studies, they bespeak an upcoming propitious tool, as they enable motivated individuals to provide their answers, rapidly, and at the touch of a button; they are automated, cost-effective, and error-free [8,15,16,17]. Primarily, the WBQ of this study consisted of binary questions for demographics such as gender, and level of education that included (i) mandatory education/high school and (ii) university education, master of science or doctor of philosophy. Considering the qualitative WBQs’ type for a better e-sample response, in addition to the fact that European countries are mainly aging, the concept of age-related questions was to follow a generation-based model with age ranges, to reveal each generation’s criticism and attitudes. Generation categories included (i) Baby Boomers (age range 57–75 in 2021); (ii) Generation X (age range 41–56 in 2021); (iii) Millennials (age range 25–40 in 2021); and (iv) Generation Z restricted in adults (18–24 in 2021). The exclusion criteria for the survey, regarding the parameter of age, were those under 18 and above 75 years of age.

For the potential influence factors, there were included 5-likert type questions that potentially influenced the final decision for COVID-19 vaccination, specifically referring to participants’ health status, their personal information from the internet, their influence specifically from television doctors-scientists, the general media presentation of COVID-19, the influence from their family, friends and work. Moreover, there were questions for the impact of the government policies for the pandemic, and, finally, the impact of their social life (referring to cafes, bar, restaurants and shopping) on their final decision. The sum of positive answers in the scale was to be presented in the survey.

Apart from these basic questions, there were some other general questions for vaccines and vaccinations. Questions for the status of childhood vaccines were included, as well as questions for the participants’ views for a personal risk for a severe COVID-19, their aspects about being more safe towards emerging pathogens with more and vaccinations, or frequent vaccinations since a vaccine induces immunity for a certain period of time. Moreover, a binary question for the participants to be afraid of a likely severe COVID-19 was included in the WBQ.

### 2.2. Population-Based Sample and WBQ Administration

The survey was conducted in the Greek mainland, and the WBQ was disseminated around late November (18–27 of November 2021) and adults were randomly invited to participate in the survey through social media shares in profiles and Facebook teams. Informed consent was obtained from all subjects during accepting participation in the study. WBQs were submitted in Google forms, and data were saved in an Excel spreadsheet.

### 2.3. Statistical Analysis

Statistical analyses were performed via the statistical software IBM SPSS statistics for Windows, Version 26.0 (headquartered in Chicago). Cronbach’s alpha coefficient was applied for internal consistency reliability. Data normality was assessed with the Kolmogorov–Smirnov test. Tests were two-tailed, and the level of statistical significance was established at *p* ≤ 0.05. Chi-square test was applied for comparisons of frequencies, and Bonferroni correction was used for comparisons between subgroups. Spearman coefficient was used to evaluate correlations between variables. The Mann–Whitney-U test and Kruskal–Wallis-H test were used for estimates of mean ranks among subgroups. Binary logistic regression analysis was considered for the investigation of potential predictors of influence in response to vaccination against COVID-19, respectively.

## 3. Results

### 3.1. The Population-Based Sample Distributed by Genders, Generations, and Education Levels

The population-based sample consisted of 5369 participants, including 1558 men (29%) and 3811 women (71%). As regards the generations, there were 771 Generation Z (14.4%), 3154 Millennials (58.7%), 1309 Generation X (24.4%) and 135 Baby Boomers (2.5%) counted in total. 1591 (29.6%) disclosed a basic education level, while 3778 (70.4%) reported to have graduated from university. Table 1 describes the distribution of genders and their education levels across the different generations.

### 3.2. Distribution of Vaccinated and Non-Vaccinated Participants by Gender, Generation, and Influence Factors

Among the participants, 3730 (69.5%) stated that they had received at least one dose of the vaccine and 1639 (30.5%) reported not to be vaccinated against COVID-19; 46.5% of the non-vaccinated disclosed that they were afraid of the vaccine, while 54% of the vaccinated people reported they were vaccinated so as not to be infected from SARS-CoV-2. Table 2 describes the distribution of vaccinated and non-vaccinated participants among genders, generations, and economic sectors.

### 3.3. The Distribution of Vaccinated Participants in Each Generation by Gender and Education Level

There was a statistically significant difference for vaccinated women except the older generation, and also, there was a significant difference for the vaccinated with university level compared to basic education in Millennials and Generation X (19.9% versus 80.1%, *p* < 0.001 and 36.1% versus 63.9%, *p* < 0.001). Table 3 shows the distribution of vaccinated individuals in each generation, between genders and by their education level.

### 3.4. Vaccinated People and Influence Factors among Genders, Generations, and Education Level

Table 4 shows the positive rates of the analyzed factors that influenced the final response for receiving a vaccine against COVID-19.

Besides, regarding the non-vaccinated participants’ responses to the same factors, the gender difference was observed only in health status (28.1% versus 35%, *p* = 0.004) and family influence (14.9% versus 19.5%, *p* = 0.020) for women. Moreover, there was no significant difference among education levels for those factors, and concerning generations, social life, work, friends, family and media had the most influence on the younger generations, as expected.

### 3.5. Investigation of Potential Predictors of Influence on Final Decision Regarding COVID-19 Vaccine

Among the participants, 5294 (98.6%) reported a positive vaccination status for the basic childhood vaccines, and 3773 (70.3%) stated that they would feel more safe towards emerging pathogens with frequent vaccinations, since a vaccine induces immunity for a certain period of time. Additionally, 3490 (65%) reported to be afraid of a possible personal severe COVID-19. A final logistic regression analysis, considering the COVID-19 vaccine status as a dependent variable, found that severe COVID-19 risk (OR 2.511; CI 2.149–2.934; *p* = 0.000), frequent vaccinations for emerging pathogens (OR 14.022; CI 11.998–16.389 and social life (OR 2.828; CI 2.417–3.309; *p* = 0.000) had a significant impact on people’s positive response to vaccination against COVID-19, as presented in Table 5.

## 4. Discussion

A Greek study revealed that, of their participants, only 57.7% stated that they are going to get vaccinated for COVID-19, mainly those aged > 65 years old, those who either themselves or a member of their household belonged to a vulnerable group, those believing that the COVID-19 virus was not developed in laboratories by humans, those believing that coronavirus is far more contagious and lethal compared to the H1N1 virus, and those believing that next waves are coming were statistically significantly more likely to be willing to get a COVID-19 vaccine, whereas higher knowledge score regarding symptoms, transmission routes, and prevention and control measures against COVID-19 was significantly associated with higher willingness of respondents to get vaccinated [14]. Another Greek study showed that about 74% of respondents supported mandatory vaccination and 62% intended to get vaccinated for COVID-19, while the most prevalent reasons against COVID-19 vaccination were safety concerns related to the duration of clinical trials and potential side effects [18].

Our study revealed that mainly women were more likely to accept vaccination, except from the older generation, whereas, generally, the older generations and those who had not graduated from university showed more hesitance to the vaccine against COVID-19. Furthermore, the vaccinated were influenced by their work, personal internet searching, and social life, while the non-vaccinated were influenced by their personal information from the internet and the government policies. The media, work, and social life showed no different impact among the education levels in the vaccinated participants. Moreover, among generations, the older vaccinated participants were more likely to be influenced by their health status, the TV doctors, and the media, in contrast with the youth, which were mostly influenced by their social life.

SARS-CoV-2 and its emerging mutants have raised global public health concern, whereas vaccination strategies have caused a decline in COVID-19 patients presented in the Emergency Departments (EDs) [19]. A study involving older adults showed that adverse events associated with Spikevax were mainly mild or moderate, and that immunogenicity was effective [20]. Despite these favourable results that are being globally announced, in our study, we found the lowest vaccination rates in the adults and especially in the older adults. Moreover, we found that women were more likely to be vaccinated than men, except from the older individuals in whom no significant difference was detected, even if the literature has highlighted that men are more likely to be vaccinated than women [21]. These results seem mournful, since COVID-19 high mortality has been correlated with men, despite the women sensitivity to vaccination status [22,23]. Despite that fact, it is now clear that mild to severe acute infection is not the only outcome of COVID-19, and long-lasting symptoms are also possible. In contrast to severe acute COVID-19, such ‘long COVID’ is seemingly more likely in women than in men [24]. Furthermore, age and comorbidities contribute to disease severity through immune-mediated mechanisms, since they are associated with a chronic increase of pro-inflammatory mediators, and cause an enhanced susceptibility to develop an immune dysregulation following SARS-CoV-2 infection [25]. A Greek study revealed the high rates of comorbidities that could lead to a severe COVID-19 distributed by generations, but, in our study, the vaccinations were reversely correlated with the generations—as compared to this study [9]. It was revealed that the youth is highly vaccinated. Thus, it is required that older generations be persuaded to get immunized against COVID-19, since they are highly presenting risk factors for a potential severe COVID-19 [9]. Moreover, university education level had an impact on vaccinated Millennials and Generation X, while the education level showed no significance in the older individuals’ vaccination status. Thus, we cannot make any further correlation for them being highly non-vaccinated.

Health status had a higher impact on the participants who decided not to get vaccinated against COVID-19, despite the societal high rates of risk factors for a severe COVID-19 [9]. However, the health status had a higher impact on the older generations that reported to be vaccinated, and this result is in parallel with the generation risk factors for a likely severe COVID-19 [9]. The personal information from the internet showed a higher impact in the non-vaccinated participants, whereas there was no significant difference in vaccinated social strata, except the education level, that the vaccinated graduates were more likely to be influenced from the internet for COVID-19. Nevertheless, the impact of the media in the COVID-19 era can have both positive and negative results, and a major limitation of social media personal use is the ability to quickly disseminate false information which can confuse and distract or even make people afraid [26]. Thus, some false information could lead some people not to get vaccinated against COVID-19. It is necessary to urge and promote the use of the websites of official public health organizations when seeking information on COVID-19 preventive measures on the internet [27]. Sadly though, misinformation, possible detrimental impacts of myths, and conspiracy theories related to COVID-19 and vaccine on COVID-19 are common for some non-vaccinated individuals [28]. Moreover, half of the vaccinated participants reported to be vaccinated so as not to be infected from SARS-CoV-2, and this result depicts the level of the general misinformation amongst vaccinated people. The expected results of a vaccination are the sufficient antibody titers, so as to prevent a likely severe COVID-19 or/and admission to the Intensive Care Unit (ICU); infection from a pathogen cannot be prevented via vaccination! Compared to the general information from the media that influenced highly the older vaccinated participants, there was an influence for the non-vaccinated individuals, and mainly the youth—as expected. However, information overload during the pandemic has posed a set of challenges not encountered before, as there is an “infodemic” in which false news, conspiracy theories, magical cures, and racist news are being shared at an alarming rate [29]. The younger generations are highly affected by such scenarios, as they are more familiar with advanced media.

Family and the workplace had no significant variations in vaccinated among generations, but these factors showed a higher influence on the positive decision to vaccination against COVID-19, compared to the negative vaccination status. This is quite logical, since it has been revealed the influence of household transmission of SARS-CoV-2, in this pandemic [30]. In contrast with that, the family had an important influence in the non-vaccinated youth. The TV scientists showed no increased rates of influence, and, in this manner, we highlight the need for media doctors–experts to be unanimous, accurate, precise, and evidence-based, so as to persuade people to accept vaccinations. The government policies had an impact on the decision of vaccinated women compared to men, the younger generation, and those with a basic education, for their positive response to the vaccine against COVID-19. However, about half of the non-vaccinated individuals were influenced by government policies in their final negative response to the vaccine. Another study showed the influence of the political parties to the final acceptance to vaccination against COVID-19 [31].

Furthermore, almost all of the participants reported a positive status for the basic childhood vaccinations. Since the induced immunity of a vaccine has a certain time duration, a high percentage of the participants reported that they would feel more safe with frequent vaccinations with new advanced vaccine technologies. However, these aspects were generally for vaccines and vaccinations, and not specified in COVID-19 vaccines. Moreover, the community itself said to be afraid of COVID-19 vaccines, in comparison to their high rates for more and frequent needed vaccines with new platforms, and this can be reasoned due to the new vaccine technologies compared to the old platforms. It is evident that recent biotechnological advances have largely overcome several issues, and multiple mRNA vaccine platforms against infectious diseases and several types of cancer have demonstrated encouraging results in both animal models and humans [32]. Moreover, the information generated by whole genome sequencing projects and the rise of bioinformatics has triggered the birth of a new era for vaccinology, leading to a “third generation” of vaccines, which are based on the application of vaccinomics science to vaccinology—the so-called reverse vaccinology [33]. Thus, the scientific community can promise favourable results for new vaccination strategies and preventive medicine’s attempts, in populaces. Nevertheless, the potential effects of frequent vaccination boosts on the immune system should be further analyzed, for this strategy to be accurate and safe. Above the need for frequent vaccines and the fear of a severe COVID-19, another significant predictor for the acceptance to vaccine against COVID-19, was the social life. It is true that non-vaccinated people face extreme limitations during this pandemic, and a percentage of them has accepted COVID-19 vaccines, due to social limitations.

Heretofore, no study is completely foolproof; we had a small population-based sample for the older individuals, the Baby Boomers. We did not include the parameter of conspiracy theories and its impact on the response to vaccination against COVID-19, and this parameter was analyzed in some other studies. Additionally, we have not included the parameter of side effects that could have an impact on the final hesitance to the vaccines, as other studies have shown [34].

## 5. Conclusions

Our study has revealed some basic factors that have influenced the final response to COVID-19 vaccination, mainly for the vaccinated individuals of our survey. Women and the younger generations were more likely to be vaccinated against COVID-19. The need for frequent vaccines for preventing severe infections from emerging pathogens, the social life, and the risk fear of a severe COVID-19 were predictors for a positive response to vaccination against COVID-19.

## Figures and Tables

**Table 1:** Genders and education level amongst generations, *n* = 5369.

Generations	*n*	Genders		*p*-Value	Education Level		*p*-Value
		Male (% Out of *n*)	Female (% Out of *n*)		Basic School (% Out of *n*)	BSc, MSc, PhD (% Out of *n*)	
Generation Z	771	173 (22.4)	598 (77.6)	0.000	202 (26.2)	569 (73.8)	0.024
Millennials	3154	878 (27.8)	2276 (72.2)	0.023	756 (24)	2398 (76)	0.000
Generation X	1309	465 (53.5)	844 (64.5)	0.000	571 (43.6)	738 (56.4)	0.000
Baby Boomers	135	42 (31.1)	93 (68.9)	0.587	62 (45.9)	73 (54.1)	0.000

**Table 2:** Vaccinated and Non-Vaccinated people among genders, generations, and the influence factors, *n* = 5369.

		Vaccinated (%) *	Non-Vaccinated (%) *	*p*-Value
Gender	Male	975 (62.6)	583 (37.4)	0.000
	Female	2755 (72.3)	1056 (27.7)	
Generations	Generation Z	581 (75.4)	190 (24.6)	0.000
	Millennials	2266 (71.8)	888 (28.2)	
	Generation X	820 (62.6)	489 (37.4)	
	Baby Boomers	63 (46.7)	72 (53.3)	
Education level	Basic education	924 (58.1)	667 (41.9)	0.000
	University Education	2806 (74.3)	972 (25.7)	
Influence factors	Health status	990 (26.5)	534 (32.6)	0.000
	Internet	1881 (50.4)	909 (55.5)	0.000
	TV scientists	1297 (34.8)	352 (21.5)	0.000
	The media	1610 (43.2)	626 (38.2)	0.001
	Family	1654 (44.3)	293 (17.9)	0.000
	Friends	922 (24.7)	195 (11.9)	0.000
	Work ^1^	1927 (51.7)	344 (21)	0.000
	Social life	1979 (53.1)	466 (28.4)	0.000
	Government	884 (23.7)	841 (51.3)	0.000

^1^: work and university for undergraduates from Generation Z. *: % within row for gender, generations, and education level, and % within column for the influence factors.

**Table 3:** Vaccinated people in each generation among genders and education level, *n* = 3730.

Generations	*n*	Genders		*p*-Value	Education Level		*p*-Value
		Male (% Out of *n*)	Female (% Out of *n*)		Basic School (% Out of *n*)	BSc, MSc, PhD (% Out of *n*)	
Generation Z	581	117 (20.1)	464 (79.9)	0.000	155 (26.7)	426 (73.3)	0.099
Millennials	2266	573 (25.3)	1693 (74.7)	0.000	452 (19.9)	1814 (80.1)	0.000
Generation X	820	266 (32.4)	554 (67.6)	0.019	296 (36.1)	524 (63.9)	0.000
Baby Boomers	63	19 (30.2)	44 (69.8)	0.841	21 (33.3)	42 (66.7)	0.518

**Table 4:** Influence factors for Vaccinated participants amongst genders, generations, and education levels, *n* = 3730.

Influence Factors	Genders		*p*-Value	Generations				Education Level		*p*-Value
	Male (% Within) *	Female (% Within) *		Generation Z (% Within) *	Millennials (% Within) *	Generation X (% Within) *	Baby Boomers (% Within) *	Basic School (% Within) *	BSc, MSc, PhD (% Within) *	
Health status	212 (21.7)	778 (28.2)	0.000	116 (20) ^a^	587 (25.9) ^b^	261 (31.8) ^c^	26 (41.3) ^c^	242 (26.2)	2058 (73.3)	0.781
Internet	497 (51)	1384 (50.2)	0.692	295 (50.8) ^a^	1147 (50.6) ^a^	413 (50.4) ^a^	26 (41.3) ^a^	404 (43.7)	1477 (52.6)	0.000
TV scientists	304 (31.2)	993 (36)	0.006	201 (34.6) ^a^	729 (32.2) ^a^	342 (41.7) ^b^	25 (39.7) ^a,b^	291 (31.5)	1006 (35.9)	0.016
The media	356 (36.5)	1254 (45.5)	0.000	261 (44.7) ^a,b^	937 (41.4) ^b^	383 (46.7) ^a^	29 (46) ^a,b^	397 (43)	1213 (43.2)	0.888
Family	393 (40.3)	1261 (45.8)	0.003	245 (42.2) ^a^	1012 (44.7) ^a^	373 (45.5) ^a^	24 (38.1) ^a^	371 (40.2)	1283 (45.7)	0.003
Friends	223 (22.9)	699 (25.4)	0.120	145 (25) ^a,b^	598 (26.4) ^b^	163 (19.9) ^a^	16 (25.4) ^a,b^	198 (21.4)	724 (25.8)	0.008
Work ^1^	478 (49)	1449 (52.6)	0.055	311 (53.5) ^a^	1139 (50.3) ^a^	446 (54.4) ^a^	31 (49.2) ^a^	480 (51.9)	1447 (51.6)	0.841
Social life	477 (48.9)	1502 (54.5)	0.003	368 (63.3) ^a^	1205 (53.2) ^b^	375 (45.7) ^c^	31 (49.2) ^a,b,c^	481 (52.1)	1498 (53.4)	0.483
Government	201 (20.6)	683 (24.8)	0.008	163 (28.1) ^a^	512 (22.6) ^b^	194 (23.7) ^a,b^	15 (23.8) ^a,b^	257 (27.8)	627 (22.3)	0.001

^1^: work and university for undergraduates from Generation Z. *: the percentage is for vaccinated participants in each column, and each subscript letter ^a,b,c^ denotes a subset of age categories whose column proportions do not differ significantly from each other at the 0.05 level.

**Table 5:** Logistic regression for the influence to the response to COVID-19 vaccination, *n* = 5369.

Variables	B	SE	Wald	Sig.	Exp(B)	95% CI for EXP (B)	
						Lower	Upper
Gender (ref. men)	−0.158	0.083	3.625	0.057	0.853	0.725	1.005
COVID-19 fear	0.921	0.079	134.387	0.000	2.511	2.149	2.934
Frequent vaccinations	2.641	0.080	1101.772	0.000	14.022	11.998	16.389
Childhood vaccines	0.602	0.311	3.745	0.053	1.826	0.992	3.359
Social life	1.040	0.080	168.098	0.000	2.828	2.417	3.309

Abbreviations: COVID-19; Coronavirus Disease 2019.

## Data Availability

Data sharing is not applicable to this article. The data are not publicly available due to restrictions. They contain information that could compromise the privacy of the participants.
